# Aging-Related Mechanisms Contribute to Corticosteroid Insensitivity in Elderly Asthma

**DOI:** 10.3390/ijms24076347

**Published:** 2023-03-28

**Authors:** Maria L. Ford, Anushka Ruwanpathirana, Brandon W. Lewis, Rodney D. Britt

**Affiliations:** 1 Center for Perinatal Research, Abigail Wexner Research Institute at Nationwide Children’s Hospital, Columbus, OH 43215, USA; maria.ford@nationwidechildrens.org (M.L.F.); anushka.ruwanpathirana@nationwidechildrens.org (A.R.);; 2 Biomedical Sciences Graduate Program, College of Medicine, The Ohio State University, Columbus, OH 43210, USA; 3 Department of Pediatrics, The Ohio State University, Columbus, OH 43205, USA

**Keywords:** aging, airway inflammation, asthma, corticosteroid insensitivity, senescence

## Abstract

Asthma in elderly populations is an increasing health problem that is accompanied by diminished lung function and frequent exacerbations. As potent anti-inflammatory drugs, corticosteroids are commonly used to reduce lung inflammation, improve lung function, and manage disease symptoms in asthma. Although effective for most individuals, older patients are more insensitive to corticosteroids, making it difficult to manage asthma in this population. With the number of individuals older than 65 continuing to increase, it is important to understand the distinct mechanisms that promote corticosteroid insensitivity in the aging lung. In this review, we discuss corticosteroid insensitivity in asthma with an emphasis on mechanisms that contribute to persistent inflammation and diminished lung function in older individuals.

## 1. Introduction

Aging is a natural process that is accompanied by changes to lung function and immunity, influencing lung disease susceptibility and progression [1]. In healthy individuals, lung function peaks around 25–30 years of age and then naturally declines [2]. This can be exacerbated by environmental exposures and comorbidities such as smoking, obesity, and diabetes [3]. Aged populations have an increased risk of developing chronic lung diseases, such as COPD and pulmonary fibrosis, and greater risk of death from infection [1]. As the number of individuals older than 65 years continues to increase in the United States and throughout the world, there is a pressing need to understand underlying mechanisms that mediate chronic lung diseases in aged populations.

As a reversible obstructive airway disease, asthma is characterized by chronic airway inflammation, airway thickening and remodeling that leads to airway narrowing and airflow obstruction. Structural changes to the airway diminish lung function and are largely irreversible despite standard of care anti-inflammatory and bronchodilator treatments [4]. The rates of asthma within aged populations are increasing, with more than 8% of people with asthma being older than 65 [5]. These increases include individuals who develop asthma early in life (early-onset asthma) and those who develop asthma as adults (late-onset asthma) [5]. Asthma symptoms in aged populations are difficult to manage due to factors that influence asthma pathogenesis and severity in older individuals including lung function decline, environmental exposures, and altered immunity [6,7]. Asthma diagnosis in older populations is further complicated by comorbidities, such as obesity and smoking, that confound asthma diagnosis and treatment.

Asthma pathogenesis involves complex innate and adaptive immune pathways that promote inflammation and may influence responsiveness to corticosteroids. Type 2 inflammation is commonly found to have increased T helper (Th) 2 effector cytokine levels (IL-4, IL-5, and IL-13) and eosinophil lung infiltration. The importance of type 2 inflammation in asthma is highlighted by the effectiveness of biologic therapies that target and inhibit IL-4 and IL-5 signaling pathways. Biologics, such as dupilumab (anti-IL4Rα) and mepolizumab (anti-IL-5), improve lung function and reduce exacerbation frequency in patients with asthma [8]. Asthma endotypes with increased type 1 or 17 inflammation have increased neutrophil infiltration and are mediated by effector cytokines, namely IFNγ and IL-17A, respectively. Recent studies show the presence of type 1 and 17 inflammation, characterized by increased neutrophils and IL-17A and IFNγ levels, are more common in older individuals with asthma [9]. These inflammatory endotypes in asthma are associated with corticosteroid insensitivity, poor symptom control, and greater asthma severity [10,11].

Although anti-type 2 biologics are safe and effective for individuals 65 and older with asthma [12,13], they are restricted to older patients with a predominant type 2 eosinophilic endotype. Additionally, those with increased type 1 and/or type 17 inflammation are unlikely to benefit from type 2 biologics, potentially making it more difficult to treat asthma in elderly populations [14]. Given the increasing aging population and limited therapeutic options, it is important to understand mechanisms that mediate asthma in the elderly. In this review, we discuss the current understanding of asthma pathogenesis in the context of aging with particular emphasis on mechanisms that may promote persistent inflammation and corticosteroid insensitivity.

## 2. Glucocorticoid Receptor Signaling and Hormones in the Aging

Corticosteroids inhibit inflammation through binding the glucocorticoid receptor (GR) and reduce pro-inflammatory pathway activation in immune and airway structural cells [15]. Upon ligand binding, GR translocates to the nucleus where it interacts with and binds DNA. GR enhances anti-inflammatory gene expression while also repressing pro-inflammatory transcriptional programs that regulate inflammation [15]. In asthma, corticosteroid insensitivity involves impaired GR signaling with increased GRβ expression, reduced GR nuclear translation, altered GR phosphorylation and DNA binding activity [16,17,18,19]. Little is known about how GR expression and activity changes with age. In the brain, studies in aged rodents have shown reduced GR expression levels in hippocampal regions [20], but it is unclear whether aging leads to reduced GR expression in the lung. In regard to aging and GR activity, one study in peripheral blood mononuclear cells reported reduced corticosteroid–GR binding affinity in cells isolated from patients 55–64 years old with severe asthma [21]. Inflammation is known to reduce corticosteroid–GR binding affinity in asthma [22], suggesting aging could affect steroid sensitivity by reducing GR expression or DNA binding activity in asthma.

Aging also has an impact on endogenous cortisol production, which can influence lung immunity and inflammatory responses. Cortisol is a circulating stress hormone that is secreted by adrenal glands and whose levels are tightly regulated [23]. The suprachiasmatic nucleus (SCN), which regulates the circadian clock, allows the release of adrenocorticotropin (ACTH) from the pituitary gland and ACTH stimulates the release of glucocorticoids from adrenal glands via the hypothalamic–corticotropic–adrenal (HPA) axis [24]. Free cortisol levels can affect metabolism and immune function in the lung [25,26]. Although old age is associated with increased free cortisol levels, it has been shown that cortisol levels are lower in children with asthma, contributing to increased inflammation and exacerbations [26,27]. These effects may be due to prolonged corticosteroid use which is known to reduce cortisol levels [28,29].

Dehydroepiandrosterone (DHEA) and DHEA sulfate (DHEAS) are also important circulating steroid hormones whose levels are inversely related to age [30]. DHEA and DHEAS are converted into androgens by 3β-hydroxysteroid dehydrogenase-1 (3β-HSD1). One study showed that DHEA supplementation reduces house dust mite-induced Th2 inflammation in mice [31]. Similarly, Dashtaki et al. found that DHEA reduced proliferation of rat tracheal smooth muscle stimulated with PDGF and inhibited AP-1 binding activity. These studies suggest that DHEA can suppress allergic airway inflammation [32]. Androgens activate androgen receptors and have recently been shown to have anti-inflammatory effects in asthma [33,34,35]. Recent studies in a severe asthma cohort found that asthma patients with a permissive 3β-HSD1 genotype that enables greater DHEA conversion into androgens was associated with better lung function and corticosteroid sensitivity [36]. These studies highlight the impact of adrenal hormones on inflammation and corticosteroid sensitivity in asthma, yet this has not been explored in aged populations. With the known impact of aging and corticosteroid treatment on adrenal hormones, such as cortisol and DHEA, it is important to consider how they change with age and may impact asthma management in elderly populations.

## 3. Asthma in the Elderly Pathogenesis

### 3.1. Airway Inflammation

Recent studies have identified distinct immune cell compositions in elderly populations that are associated with corticosteroid insensitivity and more severe asthma phenotypes. It has been shown that elderly asthmatics have greater sputum neutrophil and eosinophil levels than younger adults with asthma [37]. Elderly people with asthma also exhibit greater sputum cytokines levels, including IL-6 and IL-1β, which were both associated with increased likelihood of hospitalization due to asthma [37]. In a similar study, sputum neutrophils were greater than eosinophils in total cell number and percentage in elderly asthmatics [9]. This elderly asthma cohort also had increased IFNγ and IL-17 levels in sputum, while levels of type 2 markers, IgE and FeNO, were lower than those in younger asthma patients. The neutrophil/Th17 phenotype has been shown to be correlated with increased age and inhaled corticosteroid (ICS) dose, suggesting an impact on asthma severity in the elderly [38]. Together, these studies show elderly individuals with asthma may have an inflammatory profile that is distinct from younger individuals with asthma and may be less likely to respond to current corticosteroid and biologic treatments.

Studies in aged allergen-challenged rodents exhibit airway inflammation that is similar to that in aged humans with asthma. In response to ovalbumin or house dust mite sensitization, older mice exhibit an inflammatory milieu in the lung that is distinct from that in younger mice [39,40,41,42]. Studies show that eosinophil and neutrophil airway infiltration are greater in allergen-challenged aged mice, which is accompanied by more pro-inflammatory cytokine levels and IgE production [39,40]. Notably, studies show that aged mice have greater type 1 and 17 inflammation than young mice, exhibiting increased CD4+ Th1 and Th17 cells in the lung and spleen, and increased IFNγ and IL-17A expression levels [39,41]. The reason for enhanced Th1 and Th17 inflammation in aging and asthma remains unclear. The Th17 immune response is important for host defense at the mucosal interface [43]. In the intestinal mucosa, it has been shown that IL-17A is important for the clearance of bacterial infection [44]; thus, changes to the local environment due to reduced mucociliary clearance functions in the aged lung may play a role and lead to persistent airway inflammation. These studies implicate neutrophils as a potential contributor to airway inflammation in aging and highlight the relevance of aging rodent models to further interrogate underlying mechanisms.

Immune cells and their effector functions can be impacted by aging. Aged neutrophils are more hyperactive than younger neutrophils by exhibiting an increase in neutrophil extracellular trap release (NETosis) and higher integrin expression to aid in migration [45]. They also exhibit upregulation in several pro-inflammatory pathways, including NFκB and MAPK signaling, and cell death which is comparable to neutrophils activated by TNFα [45]. These findings raise the idea that enhanced neutrophil activation could underlie why the elderly asthmatic milieu has increased neutrophilia [46,47]. Conversely, aged asthmatic eosinophils are similar in number and presence of eosinophil-derived neurotoxin (EDN) compared to younger asthmatics. However, aged asthmatic eosinophils released less EDN upon stimulation than younger asthmatic eosinophils, thus suggesting decreased effector activity in aged asthmatic eosinophils [48]. Within this same cohort, there was an increase in sputum neutrophils in aged asthmatics but a decrease in sputum macrophages. These studies show the importance of better understanding the impact of age on not only the presence of immune cell populations but also the impact of their effector functions.

### 3.2. Airway Structure and Function

Aging is accompanied by natural decline in lung function, with forced expiratory volume (FEV_1_) peaking around 25–30 years of age [2]. Older individuals have diminished ability to clear particles from the lung and have productive coughs [49,50]. Intercostal muscle and diaphragm function are also decreased with increasing age due to the sarcopenic characteristic of aged muscles [51]. Sarcopenia is used to describe muscles that have decreased function and mass due to age. Chest expansion has also been found to be inversely related to age [52]. A productive cough requires both a high FEV_1_ and high contractile function of expiratory muscles. With the decrease of both of these functions with age, the function of the mucociliary escalator also decreases, which could contribute to increased risk of infection and alter the local immune environment [49].

It has been shown that aged human asthmatics have lower FEV_1_, higher BMIs, are less likely to be atopic, and are more likely to have other comorbidities than younger asthmatics. Elderly asthmatics are often prescribed higher doses of ICS compared to younger asthmatics [53]. The need for increased ICS dosage is often required to limit asthma symptoms and reduce exacerbation frequency [5,54]. It was also found that a higher percentage of aged asthmatics are prescribed long-acting beta agonists and add-on therapies such as leukotriene receptor antagonists than are younger asthmatics [37,53]. Aged asthmatics also exhibit decreased awareness of bronchoconstriction and recognition of asthma symptoms, which could be a possible reason for the observed increase in asthma-related deaths with age [55,56,57]. Although allergy decreases with age, the likelihood of having controlled asthma decreases with age [53], suggesting inflammatory mechanisms distinct from allergic responses contribute to asthma symptoms and exacerbations in the aging lung.

Aging also impacts lung function and airway hyperresponsiveness (AHR) in asthma. While asthma commonly affects conducting airways, airway dysfunction in elderly asthmatics is more likely to also involve peripheral airways [58]. Using impulse oscillometry (IOS), a forced oscillation technique that more accurately distinguishes the origin of lung resistance between large airways and small airways, a recent study showed that elderly asthmatics had significantly higher resistance in both large and small airways than younger asthmatics [59]. Similar findings are observed in middle-aged 9-month-old mice, who had greater airway inflammation and AHR in response to acute house dust mite challenge than younger 3-month-old mice [60]. In regard to airway thickening and remodeling, airway wall thickness is found to be decreased with age [61,62]; however, elderly asthmatics were observed to have thicker airway walls compared to nonelderly asthmatics [59]. Additionally, elderly asthmatics have higher sputum levels of pentosidine, a collagen cross-linker, than younger asthmatics [63]. These findings implicate aging in changes to airway function and structure, particularly increased airway resistance and thickening, that can affect asthma symptoms in elderly populations.

## 4. Aging-Related Mechanisms in Asthma

### 4.1. Cellular Senescence

Cellular senescence is defined by cell cycle arrest and plays a central role in aging-related diseases [49]. While senescent cells have important roles in embryonic development and homeostasis [50,51], they accumulate in aging and contribute to disease pathogenesis through secretion of inflammatory factors known as the senescence-associated secreted phenotype (SASP). Although cell- and tissue-specific, the SASP is composed of pro-inflammatory and pro-fibrotic factors that include cytokines, chemokines, proteases, and matrix metalloproteinases (MMPs), and extracellular matrix proteins [52]. Senescent cells can also secrete exosomes and ectosomes that contain microRNA, DNA fragments, chemokines, and other bioactive factors that enhance chronic inflammation [53]. In the context of lung diseases, targeting and eliminating senescent cells has been shown to reduce lung fibrosis in bleomycin-induced fibrosis in mice [54,55], suggesting senescence is a targetable pathway to alleviate lung disease.

Airway smooth muscle (ASM) is important for maintaining airway tone and function [56]. In asthma, increased ASM hypercontractility, mass, hypertrophy, and extracellular matrix protein deposition contribute to airflow obstruction and asthma exacerbation. Recent studies indicate that ASM is susceptible to aging-related mechanisms, notably cellular senescence, that promote asthma pathogenesis. Oxidative stress induced by hyperoxia increases ASM senescence and induction of a specific SASP that has autocrine and paracrine effects on the airway [57]. Increased senescence is also observed in asthmatic bronchial fibroblasts, which exhibit lower DNA synthesis with cell passage and in vitro lifespan than healthy controls [58]. Asthmatic ASM isolated from aged individuals has increased expression levels of multiple senescence markers and pathways including phospho-p53, p21, telomere-associated foci (TAF), as well as multiple SASP factors (PAI-1, TNFα, MMP1, CCL2) [59]. In a similar study, human ASM from aged individuals had greater intracellular Ca^2+^ response, fibronectin, and collagen III deposition than younger human ASM [60]. These studies suggest aged ASM may have a greater capacity for SASP production and hypercontractility that further promotes asthma pathogenesis. Despite evidence of ASM dysfunction in the aging lung, it remains unknown how corticosteroids may affect senescence-related pathways in ASM.

In addition to ASM, airway epithelial cells also play an important role in asthma [61] and are affected adversely by aging. Aged primary human airway epithelial cell cultures have reduced transepithelial resistance and altered gene expression of genes involved in epithelial barrier integrity, with increased EPCAM and decreased TRPV4 expression compared to younger cells [62]. Another protein important for airway epithelial adhesion and barrier integrity, integrin β4 (ITGB4), has been recently implicated in aging and asthma. Loss of ITGB4 in club cells in mice increases senescence in airway epithelial cells through increased p53 pathway signaling [63]. Similarly, club cell-specific ITGB4 knockout mice challenged with house dust mite develop greater airway thickening and remodeling with more collagen deposition and mucous cell metaplasia [64]. Interestingly, treatment with the corticosteroid dexamethasone reduced inflammation and remodeling in wild-type but not club cell-specific ITGB4 knockout mice [64]. While the airway epithelial SASP is not defined for asthma, recent studies reported altered WNT/β-Catenin signaling between bronchial epithelium and fibroblasts that increased fibroblast senescence and airway remodeling [65]. These data suggest that aging may negatively impact the airway epithelium and disruption to changes to adhesion and barrier integrity can promote senescence and persistent airway remodeling in asthma.

**Figure 1:**
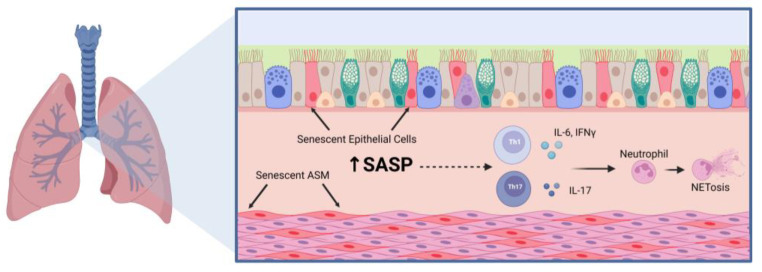
Notable Inflammatory Mechanisms in the Aged Asthmatic Lung. The aged asthmatic airway has persistent airway inflammation that includes increased senescent epithelial and airway smooth muscle (ASM) cells, which secrete pro-inflammatory and pro-fibrotic factors that contribute to airway inflammation. The senescence-associated secretory phenotype (SASP) can include IL-1β, IL-6, IL-8, TNFα, matrix metalloproteinases, and extracellular matrix proteins such as fibronectin and collagen. Among these inflammatory mediators are cytokines that are known to promote T helper (Th) 1 and Th17 cell differentiation and effector functions which can promote neutrophil infiltration and activation. Figure was created using BioRender.com accessed on 7 March 2023.

Currently, there are very few studies that prove a clear connection between aging, senescence, and skewing to Th1 and Th17 inflammation. Studies in middle aged (8–12-month-old) mice showed increased T helper cell skewing towards the Th1 phenotype with observed increases in IFNγ in allergen-challenged mice [66]. This was further emphasized with a decrease in Th2 differentiation and effector IL-4 and IL-13 production, which was attributed to decreased GATA-3 expression. In regard to corticosteroid sensitivity and aging, Jaiswal et al. suggested that airway inflammation persists in aged mice treated with corticosteroids, and further explore this relationship [39]. Upon house dust mite challenge, dexamethasone decreased macrophage and IL-13 levels in 80–82-week-old mice but neutrophil infiltration along with IL-17A, IFNγ, and IgE levels remained increased and higher than those in 20–22-week-old mice. Aside from persistent airway inflammation, it was also observed that aged mice had persistent airway remodeling that was not resolved with the addition of dexamethasone. Aged mice challenged with house dust mite also had increased p16+ senescent airway epithelial cells that produced SASP factors including MMP10, MMP12 and TGFβ. Aged epithelial cells and dendritic cells also had increased β-galactosidase+ cells, suggesting there is an increase in senescence. Through these studies, it can be proposed that the SASP contributes to inducing skewing to Th1 and Th17 inflammation, leading to corticosteroid insensitivity (Figure 1). This reveals a possible connection between the aging phenotype and the inflammatory phenotype of the aged asthmatic lung.

### 4.2. CD38 and NAD^+^ Metabolism

**Figure 2:**
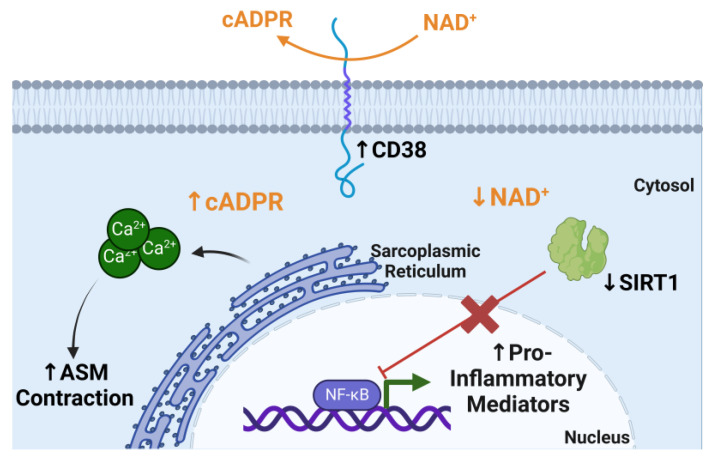
CD38 NAD^+^ metabolism increases cADPR levels and limits SIRT1 activity. CD38 consumes nicotinamide adenine dinucleotide (NAD^+^) to convert it to cyclic adenosine diphosphate ribose (cADPR) which is used intracellularly to induce calcium (Ca^2+^) release from the sarcoplasmic reticulum, which allows the contraction of airway smooth muscle (ASM). Sirtuin 1 (SIRT1) inhibits NFκB activation; however, this consumption of NAD^+^, the SIRT1 cofactor, limits SIRT1 deacetylase activity, which allows the production of pro-inflammatory mediators via NFκB activation. Figure was created using BioRender.com accessed on 20 March 2023.

Cluster of differentiation 38 (CD38) is a glycoprotein that is expressed by ASM in addition to other cells, including epithelial cells, dendritic cells, T and B cells, and macrophages [67]. CD38 expression and activity has also been shown to increase with age and is implicated in aging-related diseases [68,69]. CD38 cyclase activity converts nicotinamide adenine dinucleotide (NAD^+^) into cyclic adenosine diphosphate ribose (cADPR), a calcium-signaling second messenger which is essential for ASM contraction (Figure 2) [70]. CD38 is the main consumer of NAD^+^, which coincides with the intuitive decrease in NAD^+^ levels with age [69]. In the context of asthma, CD38 promotes ASM hypercontractility and AHR [70]. Notably, CD38 expression induced by type 1 pro-inflammatory cytokines, TNFα and IFNγ, in human ASM is insensitive to corticosteroids [71,72]. Upon acute allergen challenge, adult CD38 knockout mice have decreased airway hyperresponsiveness compared to allergen-challenged wild-type mice [73,74,75,76]. Recent studies by Cui et al. found increased CD38 expression in idiopathic pulmonary fibrosis lungs and an important role for alveolar epithelial cell CD38 in a bleomycin lung fibrosis aged mouse model [77]. Increased CD38 expression corresponded with reduced NAD^+^ levels. With this, it can be speculated that increased CD38 expression and activity can further augment the aged asthmatic phenotype. However, there is still a need to study the role of CD38 in the context of aging and corticosteroid insensitivity in asthma.

The impact of CD38 metabolism on NAD^+^ also has an impact on epigenetic and transcriptional regulation in aging [69]. NAD^+^ is a master regulator of many metabolic functions, including redox reactions, energy metabolism, and DNA repair [69]. One example is Sirtuin 1 (SIRT1), which uses NAD^+^ as a substrate for its deacetylase activity on histones and other target proteins. Increased CD38 activity and NAD^+^ metabolism reduces SIRT1 deactylase activity (Figure 2). In the context of asthma, SIRT1 is thought to play an anti-inflammatory role by regulating Th2 cell differentiation and inhibiting pro-inflammatory pathways such as NFκB [78]. Additionally, SIRT1 expression levels and activity have been found to be decreased in asthma and COPD patients [79,80]. Changes in epigenetic regulation are very closely related to aging and often age-related epigenetic changes lead to genomic instability [81]. The impact of CD38 on NAD^+^ metabolism and sirtuins is an example of an aging-related mechanism that could contribute to airway inflammation in asthma (Figure 2). It will be important to continue to identify metabolic and inflammatory mechanisms that contribute to highlighting asthma pathophysiology in elderly populations.

## 5. Factors and Comorbidities That May Influence Asthma in the Elderly

Aged asthmatics are more likely to have comorbidities than younger asthmatics, which often complicates asthma diagnosis and disease management [82]. These comorbidities can include cardiovascular disease, obesity, and chronic obstructive pulmonary disease (COPD) [83]. Asthma patients with more than one comorbidity are more likely to have difficult-to-treat asthma, suggesting an influence of aging-related comorbidities on asthma severity [84]. One study specifically found ischemic heart disease, but not stroke, was positively correlated with older asthmatics but not younger asthmatics [85]. Additionally, it has been observed that those with persistent asthma have an increased cardiovascular disease risk compared to those with intermittent asthma [86].

Obesity is another comorbidity that is associated with corticosteroid insensitivity and poor control of asthma symptoms [87,88]. Asthma patients with obesity present with a distinct inflammatory endotype that includes increased IL-6, Th17 inflammation, and neutrophil infiltration [88,89,90]. Lung function is also worsened by obesity with increased airflow obstruction and airway hyperresponsiveness [91,92]. Studies in human ASM isolated from obese individuals exhibit greater intracellular Ca^2+^ and contractile responses that are attributed to increased myosin light chain phosphorylation and ASM shortening [93]. Metabolic dysfunction is another important factor that could affect asthma. Metabolic dysfunction and insulin resistance can be caused by obesity and were recently found to be associated with worsened lung function in a severe asthma cohort [94]. Severe asthma patients with insulin resistance had increased blood neutrophil levels and were poor responders to corticosteroids and β-agonists [94]. Mice fed a high-fat diet for several weeks develop metabolic dysfunction and AHR [95]. In addition to impaired lung function, these mice exhibit increased IL-17A levels that were dependent upon innate lymphoid type 3 cells and inflammasome activation in macrophages [95]. These studies suggest obesity can induce nonallergic airway inflammation that is mediated by innate immune responses. Although similar pathways are observed in elderly patients with asthma, additional studies in aged mice are required to identify nonallergic inflammatory mechanisms that promote asthma symptoms.

As a leading risk factor for COPD, cigarette smoke induces oxidative stress and lung inflammation that includes Th17 inflammation and senescence [96,97]. Smoking history is common in elderly individuals with asthma, making it an important influencing factor in asthma pathogenesis [9]. Asthma–COPD overlap (ACO), a syndrome with persistent airflow obstruction and distinct clinical presentation, is an additional consideration that may be important to understanding asthma in aging populations [98,99]. These patients tend to be older, have a >10 pack-year smoking history, and require use of inhaled corticosteroids at relatively high doses, suggesting corticosteroid insensitivity [99]. In a new mouse model of ACO, female mice were exposed to house dust mite and cigarette smoke for 11 weeks [100]. ACO mice had increased immune cell infiltration, with eosinophils and neutrophils, and AHR. Interestingly, cigarette smoke did not cause house dust mite-induced allergic airway inflammation as evidenced by the absence of an increase in serum IgE levels, suggesting an impaired allergen sensitization response. RNA-seq analysis of airway and parenchyma tissue identified a transcriptional profile that was unique to combined cigarette smoke and house dust mite exposure. While treatment with dexamethasone was largely ineffective, inhibition of the transcription factor SPI1 Proto-oncogene led to reduced collagen deposition and AHR in ACO mice [100]. While these studies were not performed in aged mice, they do suggest a potentially unique interaction between allergic airway inflammation and cigarette smoke exposure that may affect asthma in aged populations.

Given the complex biology in the aging lung and throughout the body, it will be important to consider how additional factors and comorbidities, such as sex hormones, impact asthma in aged populations. In addition to cortisol and DHEA, the levels of sex hormones, such as estrogen and testosterone, change with age and undoubtedly impact the development of late-onset asthma [101,102]. In a recent meta-analysis, it was shown that the proportion of adult females categorized as having late-onset asthma was higher than that of adult males [103]. This increased prevalence can be attributed to cyclical changes in estrogens, which regulate immune cell function and airway structural cell function [102,104]. However, the influence of sex hormones on corticosteroid sensitivity in the context of asthma in the elderly has yet to be explored.

## 6. Conclusions

The corticosteroid-insensitive, aged asthmatic phenotype has been well characterized, with high Th17-associated inflammation and neutrophilia along with reduced lung function [5,9]. Yet little is known about the underlying mechanisms that lead to the persistence of this potentially distinct phenotype despite corticosteroid treatment. Here, Th17 inflammation, cellular senescence, and aging-related changes to NAD^+^ metabolism appear to have important roles (Table 1), but there are likely additional mechanisms involved. Some unanswered questions include the following: (1) Why does the inflammatory response skew towards Th17 inflammation?; (2) What additional cellular mechanisms contribute to dysfunction in airway structural cells?; and (3) Can alternative anti-inflammatory strategies be developed to improve corticosteroid sensitivity? Our review highlights opportunities for leveraging age-appropriate mouse models and primary lung cell models to study aging-related mechanisms in asthma to further address these important questions.

Although the clinical presentation and characterization of elderly asthma has increased in recent years [5,9,37], there is still a wide knowledge gap in understanding the underlying mechanisms related to asthma in older individuals. Persistent inflammation and corticosteroid insensitivity among inflammatory pathways in immune and airway structural cells may be a defining feature in asthma in the elderly. Understanding the unique manifestation of elderly asthma can aid physicians in more effectively managing asthma and could lead to better therapeutic options. For additional insight into asthma management in elderly populations, we refer the reader to recent articles [105,106,107]. With the aging population continuing to increase, research efforts to understand asthma in the elderly and improve corticosteroid sensitivity remain important and need to be addressed by basic, translational, and clinical research.

**Table 1:** Mechanisms of Asthma in the Elderly.

Mechanisms	Characteristics	References
Hormone Production	Decreased Free Cortisol Decreased DHEA	[25,26,27,28,29]
Immune Cell Infiltration	Type 1/17 Inflammation Increased Neutrophil Infiltration Increased Neutrophil Activity Decreased Eosinophil Degranulation	[38,45,48]
Lung Function	Decreased FEV_1_ Increased AHR Increased Collagen Deposition	[82,108,109]
Cellular Senescence	Increased SASP factors (MMPs, TNF_α_, CCL_2_) in Airway Smooth Muscle and Epithelium Reduced ITGB4 expression	[39,52,59,63,64]
NAD^+^ Metabolism	Decreased NAD^+^ Levels Increased CD38 Expression Decreased SIRT1 Levels	[68,69,79,80]

## Data Availability

Not applicable.
